# miR-302d Targeting of CDKN1A Regulates DNA Damage and Steroid Hormone Secretion in Bovine Cumulus Cells

**DOI:** 10.3390/genes14122195

**Published:** 2023-12-10

**Authors:** Jianbo Liu, Jiabao Zhang, Yi Zheng, Guokun Zhao, Hao Jiang, Bao Yuan

**Affiliations:** 1 Department of Laboratory Animals, College of Animal Sciences, Jilin University, Changchun 130062, China; liu8541@126.com (J.L.); zjb@jlu.edu.cn (J.Z.); zhaogk20@mails.jlu.edu.cn (G.Z.); jhhaojiang@jlu.edu.cn (H.J.); 2 Experimental Testing Center, Jilin Agricultural Science and Technology University, Jilin 132101, China

**Keywords:** bovine, cumulus cells, CDKN1A, miR-302d, DNA damage, steroid hormone

## Abstract

(1) Background: DNA damage in cumulus cells hinders oocyte maturation and affects steroid hormone secretion. It is crucial to identify the key factors that regulate cellular DNA damage and steroid hormone secretion. (2) Methods: Treatment of bovine cumulus cells with bleomycin to induce DNA damage. The effects of DNA damage on cell biology were determined by detecting changes in DNA damage degree, cell cycle, viability, apoptosis, and steroid hormones. It was verified that mir-302d targeted regulation of CDKN1A expression, and then affected DNA damage and steroid hormone secretion in cumulus cells. (3) Results: Bleomycin induced increased DNA damage, decreased G1-phase cells, increased S-phase cells, inhibited proliferation, promoted apoptosis, affected E_2_ and P_4_ secretion, increased *CDKN1A* expression, and decreased miR-302d expression. Knockdown of *CDKN1A* reduced DNA damage, increased G1-phase cells, decreased G2-phase cells, promoted proliferation, inhibited apoptosis, increased E_2_ and P_4_ secretion, and increased the expression of *BRCA1*, *MRE11*, *ATM*, *CDK1*, *CDK2*, *CCNE2*, *STAR*, *CYP11A1*, and *HSD3B1*. The expression of *RAD51*, *CCND1*, *p53*, and *FAS* was decreased. Overexpression of CDKN1A resulted in the opposite results. miR-302d targets *CDKN1A* expression to regulate DNA damage and then affects the cell cycle, proliferation, apoptosis, steroid hormone secretion, and the expression of related genes. (4) Conclusions: miR-302d and CDKN1A were candidate molecular markers for the diagnosis of DNA damage in bovine cumulus cells.

## 1. Introduction

Cumulus cell–oocyte complexes (COCs) are recognized as the functionally responsible unit of the germ cell in female mammals [1]. Gap junctions between cumulus cells (CCs) and oocytes modulate the expression of hormonal factors and related regulators in oocytes, thereby affecting oocyte maturation [2]. DNA damage can be caused by diseases or environmental changes [3]. Before ovulation, oocytes need to maintain corresponding physiological functions for a long time in the body and will accumulate some damage, including DNA damage. CCs have a protective effect on oocytes and can reduce damage to oocytes from harmful substances during oocyte maturation. The expression of genes in CCs is highly correlated with the developmental competence of oocytes, mainly in DNA damage, cell cycle, apoptosis, metabolism, meiosis, and cell signaling pathways [4,5,6]. Animals need an efficient DNA damage recognition and repair (DDR) mechanism to ensure the reproductive capacity of oocytes in the ovary. Failure to properly activate DDR mechanisms can result in an inability of oocytes to mature and fertilize properly, leading to diseases or mutations in offspring [7,8]. After DNA damage occurs, chromatin reorganization, cell cycle arrest, apoptosis, or other forms of cell death may occur if the DDR is not activated correctly [9,10,11]. DNA damage in CCs could inhibit the resumption of oocyte meiosis through gap junctions [12]. DNA damage has been linked to abnormal levels of estrogen(E_2_) and progesterone(P_4_) in women [13].

MicroRNAs (miRNAs) play a critical role in the post-transcriptional modulation of gene expression and have become a focus of research on reproductive regulation mechanisms [14]. Studies have shown that DNA damage is regulated by miRNAs [15]. miRNA affects estradiol and progesterone secretion in CCs [16,17]. The abnormal expression of miR-302d is related to the productivity, migration, and apoptosis of endometrial cancer cells [18]. miR-302d can affect the cell viability and apoptosis of chondrocytes by regulating target genes [19]. Earlier studies reported that CDKN1A is a protein of 165 amino acids and belongs to the CIP/Kip group of CDK inhibitors [20]. The carboxyl group of CDKN1A reacts with PCNA to inhibit DNA damage [21]. Mammalian cell cycle progression is regulated by CDKs and the regulatory subunit cyclins, cell cycle progression is triggered by partial phosphorylation of Rb by CDK-Cyclin, and CDKN1A disrupts this interaction and affects cell cycle progression and cell proliferation [22,23]. Earlier studies showed that miR-302d and CDKN1A were abnormally expressed during DNA damage in bovine cumulus cells [24]. This indicated that CDKN1A and miR-302d may be potential markers of DNA damage. However, it is unclear whether miR-302d or CDKN1A is involved in the regulation of DNA damage and steroid hormone production in bovine cumulus cells. Bleomycin (BLM) is a chemotherapeutic agent that is often used to induce DNA damage in cells [25]. After DNA damage, γH2AX is immediately recruited to the lesion site and is the most effective biomarker to detect DNA damage [26]. Therefore, this study investigated the regulatory effects of miR-302d and CDKN1A on DNA damage in bovine cumulus cells and their effects on steroid hormone secretion.

## 2. Materials and Methods

### 2.1. Collection and Culture

Collection and culture of bovine CCs were performed in the same way as previously described [24]. In short, the ovaries were taken from healthy cows from Changchun Haoyue Halal Meat Products Co., Ltd.* (Changchun, Jilin, China) and transferred to the laboratory within 1 h. Follicular fluid containing COCs in follicles 3–8 mm in diameter was obtained using a 10 mL syringe. After three washes with HEPES (Gibco, Paisley, Scotland, UK), more than five layers of tightly packed CCs were collected under a microscope. CCs were subsequently isolated from oocytes using 0.1% hyaluronidase. CCs were then collected after centrifugation for 5 min and washed twice with phosphate-buffered saline (PBS). CCs were then cultivated in DMEM/F12 (Gibco, Grand Island, NY, USA). The culture medium was supplemented with 1% penicillin, streptomycin (HyClone, Logan, UT, USA), and 10% fetal bovine serum (Biological Industries, Kibbutz Beit Haemek, Israel), and placed in an incubator at 38.5 °C under 5% CO_2_.

### 2.2. Cumulus Cell Treatment

After CCs were cultured in 6-well plates (5 × 10^5^ cells/well) for 6 h, the original culture medium was removed. A culture medium containing 0 μM BLM or 200 μM BLM (Thermo, Carlsbad, CA, USA, diluted with cell culture medium) was added to the culture medium and treated for 3 h.

### 2.3. Cell Transfection

After culturing the cells in a 6-well plate with 70% cell fusion, 100 nM si-*CDKN1A* was transfected for the *CDKN1A* knockdown assay in accordance with the instructions of the RiboFECT CP Transfection Reagent (Ribo, Guangzhou, China). Cells were transfected with 500, 1000, and 1500 ng/mL *CDKN1A* overexpression plasmids for the *CDKN1A* overexpression assay. miRNA regulation experiments were performed by transfecting 100 nM miR-302d mimics or miR-302d inhibitor. The specific operations were as follows: (a) Diluted si-*CDKN1A*, *CDKN1A* overexpression plasmid, or miR-302d mimics/inhibitor were mixed with RiboFECT CP Transfection Reagent (Ribo, China), and the mixture was incubated indoors for 15 min. (b) The transfection mixture was added dropwise to the cells, and the plates were incubated in an oven at 38.5 °C and 5% CO_2_ for 48 h (miRNA-24 h). siRNA, overexpression plasmids, or miR302d mimics/inhibitor were synthesized by GenePharma (GenePharma, Suzhou, China). For the siRNA sequences, see Supplementary Table S1.

### 2.4. RNA Extraction and RT–qPCR

After transfection or treatment of CCs, total RNA was isolated with TriPure (Roche, Basel, Switzerland), and different reagents were used according to the manufacturer’s instructions. All RNA samples were tested for integrity and purity. An Agilent 2100 device (Agilent, Karlsruhe, Baden-Württemberg, Germany) was used to detect integrity, and a NanoDrop ND-2000 Spectrophotometer (Thermo, Waltham, MA, USA) was used to detect concentration and purity. RNAs with integrity meeting RIN value ≥ 8.0, 28S/18S ≥ 1.5, normal 5S peak, and purity meeting OD 260/280 ≥ 1.8 and OD 260/230 ≥ 2.2 were used for further experiments. Reverse transcription of RNA was performed with a reverse transcription kit (Tiangen, Beijing, China), which was used for RT–qPCR. Power SYBR Green (Tiangen, China) was used for real-time PCR assays. The overall 20 μL RT–qPCR mixture consisted of 8 μL deionized water, 10 μL SuperReal PreMix Plus (Tiangen, China), 1 μL of cDNA, and 0.5 μL of forward and reverse primers (10 mM). All real-time PCR programs consisted of 120 s of denaturation at 95 °C and 45 cycles (15 s of denaturation at 95 °C, 15 s of annealing at 60 °C, and 30 s of extension at 72 °C). Treatments were repeated at least three times in each group. Comparative expression was calculated and normalized by the 2^−ΔΔCt^ method with respect to *GAPDH (miRNA-U6)*. The sequences of the primers used are shown in Supplementary Table S1.

### 2.5. γH2AX Detection

After transfection or treatment, 500 μL of trypsin was used to dissociate the cells for 3 min. Cells were collected in a 1.5 mL centrifuge tube, then centrifuged at 500 g for 5 min and washed three times with PBS. The cells were then fixed with 100 μL (4%) paraformaldehyde solution for 10 min. Next, 900 μL of pre-cooled methanol was directly added, mixed gently, and incubatedon ice for 30 min. After centrifugation, the solution was removed, and 1 mL of incubation solution (500 g BSA in 100 mL PBS) was added to the centrifuge tube and mixed well. The mixture was centrifuged at 500× *g* for 5 min and the incubation solution was removed. Cells were treated with 100 μL γH2AX antibody (CST, Danvers, MA, USA) diluent for 1 h at room temperature. The ratio of antibody to incubation solution was 1:50. Afterwards, the cells were washed three times with incubation solution. Finally, cytosolic cells were resolved and analyzed in 200 μL of PBS with the use of a flow cytometer (CytoFLEX, Beckman Coulter, Brea, CA, USA).

### 2.6. Cell Proliferation Assays

A CCK-8 kit (Beyotime, Shanghai, China) was used to measure the proliferation of bovine CCs. Briefly, 96-well plates contained 1 × 10^4^ CCs per well in 100 μL of medium and were incubated at 38.5 °C and 5% CO_2_ for 12 h. After culturing the bovine CCs for 24 h, the cells were treated for 0 h, 24 h, 48 h, or 72 h. A solution of 10 µL CCK-8 was then injected into every well, and the incubation was carried out at 38.5 °C for 3 h. An enzyme marker (BioTek Instruments, Winooski, VT, USA) was used to measure absorbance at 450 nm.

### 2.7. Cell Cycle Analysis

Determination of the cell cycle was performed under strict adherence to the instructions of the Cycle Kit (Beyotime, China). In brief, 6-well plates were inoculated with 5 × 10^5^ CCs per well. Following transfection or treatment, CCs were collected into 1.5 mL centrifuge tubes and centrifuged at 500× *g* over 5 min. The cells were frozen in 70% ethanol for 24 h. After cleaning with PBS, the cells were re-suspended in 500 μL of dye buffer containing PI (50 μg/mL) and RNaseA (1 mg/mL) and incubated at 37 °C for 30 min away from light. The suspension was resuspended in 100 μL of PBS. Cell cycle distributions were detected in a flow cytometer (CytoFLEX, Beckman, USA), and data were processed using MODFIT software (v. 2.0, Verity Software House, Topsham, ME, USA).

### 2.8. Apoptosis Analysis

An assay for apoptosis was performed in strict accordance with the directions of the Apoptosis Detection Kit (Beyotime, China). Bovine CCs (5 × 10^5^ cells per well) were inoculated into 6-well plates. After treatment, in each centrifuge tube, 100 μL of PBS was added, followed by 5 μL of FITC solution and 5 μL of propidium iodide (20 μg/mL). It was then grown in the shade at room temperature for 15 min. Measurement of samples was performed within 2 h by a flow cytometer (CytoFLEX, Beckman Coulter, USA). Propidium iodide and FITC staining were used to distinguish apoptotic cells from dead cells.

### 2.9. Analysis of Steroid Hormone Secretion

After the cells were transfected, the levels of estradiol or progesterone secreted by bovine cumulus cells were measured by ELISA kits (Elabscience, Wuhan, Hubei, China). Briefly, (a) 100 μL of cell culture solution was centrifuged at 500× *g* over 5 min, and the supernatant was placed in a centrifuge tube. (b) An amount of 50 μL standard solution of different concentrations per well was added to the standard well. (c) A total of 100 μL 1 × HRP (10 μL 100 × HRP concentrate + 990 μL diluent)-labeled estradiol or progesterone antibody was added to the sample wells and standard wells and incubated at 37 °C for 1 h. (d) The liquid was emptied from each reaction hole, and 50 μL of substrate A and B were added to each reaction well and incubated at 37 °C for 15 min under dark conditions. (e) Fifty microliters of termination solution were added to every reaction well, and the OD value of each reaction well was measured at 450 nm within 15 min using an enzyme-labeling instrument (BioTek Instruments, USA).

### 2.10. DualLuciferase Reporter Gene Analysis

Within the dual luciferase reporter gene test, the *CDKN1A*3′UTR was cloned and inserted into the pmiR-RB REPORT™ plasmid to construct the primr-CDKN1A-3′UTR-WT plasmid. Next, miR-302d mimics and NC, primr-CDKN1A-3′UTR-WT plasmid, or pmiR-RB REPORT™ plasmid were transfected into cumulus cells. CCs were cultured in 96-well plates (1 × 10^4^ cells/well) and transfected with Ribo transfection reagent (Ribo, China) over 48 h. Then, the fluorescence intensity was measured by a fluorescence microplate reader. Reporter plasmid construction and luciferase reporter gene assays were performed by Guangzhou Ribo Biotechnology Co., Ltd. (Guangzhou, China, www.ribobio.com, accessed on 1 December 2023).

### 2.11. Statistical Analysis

Statistical analysis was performed with SPSS 23.0 (IBM, Armonk, NY, USA) [27]. Data analysis between the two groups used an unpaired *t*-test. Data were shown as mean ± standard deviation (SD). Significant differences are denoted by * (*p* ≤ 0.05) and ** (*p <* 0.01).

## 3. Results

### 3.1. Effect of DNA Damage on Cumulus Cells in Bovines

**Figure 1:**
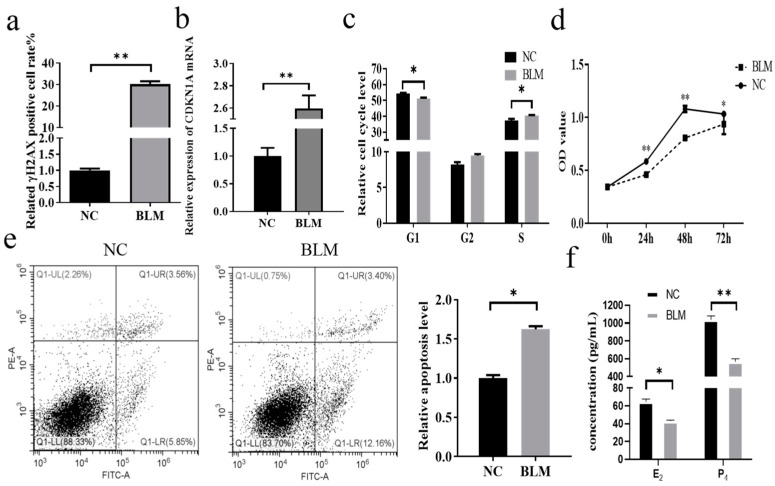
Effect of DNA damage on bovine cumulus cells. Significant differences are denoted by * (*p* ≤ 0.05) and ** (*p* < 0.01). After 200 μM BLM treatment of bovine cumulus cells for 3 h, the γH2AX-positive cell rate, cell cycle, and apoptosis were detected by flow cytometry; cell proliferation by CCK-8; *CDKN1A* expression by RT–qPCR; and E_2_ and P_4_ by ELISA. (**a**) The rate of γH2AX-positive cells was significantly higher than that in the NC after BLM treatment (*p* ≤ 0.05). (**b**) DNA damage caused a marked rise in *CDKN1A* mRNA expression (*p* ≤ 0.05). (**c**) After DNA damage, compared to the NC group, the BLM group had fewer cells in the G1 phase (*p* ≤ 0.05), clearly more cells in the S phase (*p* ≤ 0.05), and more cells in the G2 phase, but the difference was not marked (*p* > 0.05). (**d**) After the onset of DNA damage, the cell proliferation levels at 24 h (*p* < 0.01), 48 h (*p* < 0.01), and 72 h (*p* ≤ 0.05) in the BLM group were significantly lower than those in the NC group, while the difference was not significant at 0 h (*p* > 0.05). (**e**) The relative apoptotic level of cells was significantly increased after DNA damage occurred (*p* ≤ 0.05); left upper/Q1-UL: dead cells, right upper/Q1-UR: advanced apoptotic cells, right lower/Q1-LR: earlier apoptotic cells, left lower/Q1-LL: nonexpired cells. (**f**) After DNA damage occurred, the E_2_ and P_4_ contents in the cell cultures of the BLM group were significantly reduced relative to those of the NC group (*p* ≤ 0.05).

In this study, a flow cytometry assay after treating bovine cumulus cells with 200 μM BLM revealed that the rate of γH2AX-positive cells was observed to be higher in the BLM group than in the NC group (*p* < 0.01, Figure 1a). Since γH2AX is a marker of DNA damage, BLM successfully induced DNA damage in cumulus cells. In this study, the changes in CDKN1A expression after DNA damage occurred in the cells were analyzed by RT–qPCR, and we found that the expression of *CDKN1A* was significantly higher in the BLM group than in the control group (*p* < 0.01), which was in accordance with the results of a previous study (Figure 1b). We clarified the variations in the proliferation and cell cycle of CCs after DNA damage occurred. In this study, after treating the cells with BLM, a flow cytometry assay revealed that the CCs in the BLM group passed through the G1 phase rapidly and were significantly blocked in the S phase (*p* ≤ 0.05), but no significant difference was observed in the G2 phase (*p* > 0.05, Figure 1c). CCK-8 detection demonstrated that the cell proliferation level in the BLM group was obviously lower than that in the NC group at 24 h (*p* < 0.01), 48 h (*p* < 0.01), and 72 h (*p* ≤ 0.05) after DNA damage occurred (Figure 1d). Flow cytometry detection of apoptosis revealed that the relative apoptosis level in the NC group was notably lower than that in the BLM group (*p* ≤ 0.05, Figure 1e). To verify the effect of DNA damage on steroid hormone secretion in cumulus cells, the levels of E_2_ and P_4_ in cell cultures were measured by ELISA. The results showed that the secretion of E_2_ and P_4_ in cumulus cells was significantly reduced after the occurrence of DNA damage (*p* ≤ 0.05, Figure 1f, please check raw data in Table S2).

### 3.2. Effect of CDKN1A on DNA Damage and Steroid Hormone Secretion in Bovine Cumulus Cells

**Figure 2:**
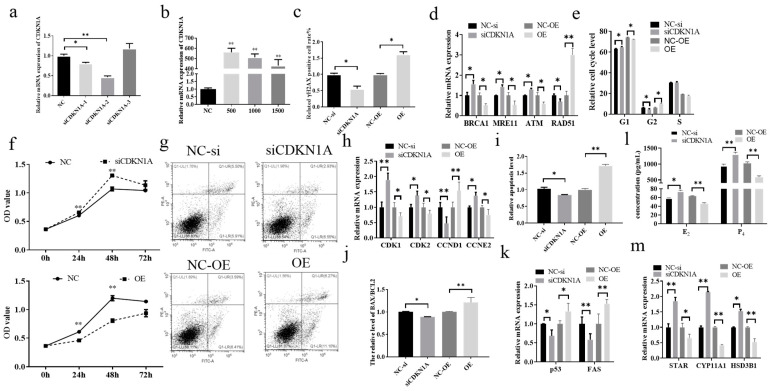
Effect of DNA damage on bovine cumulus cells. Significant differences are denoted by * (*p* ≤ 0.05) and ** (*p* < 0.01). After knocking down or overexpressing *CDKN1A*, the cell cycle, γH2AX-positive cell rate, and apoptosis were detected by flow cytometry; cell proliferation was detected by CCK-8; E_2_ and P_4_ content in cell culture medium was detected by ELISA; and related gene expression was detected by RT–qPCR. (**a**) After siRNA interference of the cells, the siCDKN1A-1 group (*p* ≤ 0.05) and siCDKN1A-2 (*p* < 0.01) group showed a marked decrease, and the siCDKN1A-3 group showed an increase (*p* > 0.05). (**b**) The expression level of *CDKNA1* substantially rose after transfection with the overexpression plasmid (*p* < 0.01), and CDKNA1 expression was the highest in group 500. (**c**) The γH2AX-positive cell rate was significantly reduced after knocking down *CDKN1A* in cumulus cells. After overexpression of *CDKN1A*, the γH2AX-positive cell rate (*p* ≤ 0.05) was significantly increased. (**d**) Inhibition of *CDKN1A* promoted the expression of *BRCA1*, *MRE11*, and *ATM* and suppressed the expression of *RAD51* (*p* ≤ 0.05). After overexpression of *CDKN1A*, the expression of *BRCA1*, *MRE11*, and *ATM* was suppressed (*p* ≤ 0.05), while *RAD51* expression was markedly increased (*p* < 0.01). (**e**) Transfection with siCDKN1A markedly increased the number of cells in G1 phase (*p* ≤ 0.05), significantly declined the number of CCs in G2 phase (*p* ≤ 0.05), and minimized the number of cells in S phase, but the change was not significant (*p* > 0.05). Overexpression of *CDKN1A* declined the number of cells in G1 phase and aggrandized the number of cells in G2 phase (*p* ≤ 0.05), and the change in S phase was not marked (*p* > 0.05). (**f**) After *CDKN1A* was knocked down, the cell proliferation level at 24 h and 48 h was significantly higher than that of the NC group (*p* < 0.01), and the difference between 0 h and 72 h was not significant (*p* > 0.05). *CDKN1A* overexpression significantly inhibited cell proliferation at 24 h and 48 h (*p* < 0.01), and the variations between 0 h and 72 h were not significant (*p* > 0.05). (**g**) The results of the apoptosis level assay. After transfection of cells, left upper/Q1-UL: dead cells, right upper/Q1-UR: advanced apoptotic cells, right lower/Q1-LR: earlier apoptotic cells, left lower/Q1-LL: nonexpired cells. (**h**) Inhibition of *CDKN1A* significantly promoted the expression of *CDK1* (*p* < 0.01), *CDK2* (*p* ≤ 0.05), and *CCNE2* (*p* ≤ 0.05) and significantly inhibited the expression of *CCND1* (*p* ≤ 0.05). After overexpression of *CDKN1A*, the expression of *CDK1*, *CDK2*, and *CCNE2* was substantially reduced (*p* ≤ 0.05), and *CCND1* expression was considerably increased (*p* < 0.01). (**i**) Inhibition of *CDKN1A* markedly declined the level of apoptosis (*p* ≤ 0.05). The relative apoptotic level of cells was markedly enhanced after overexpression of *CDKN1A* (*p* < 0.01). (**j**,**k**) Inhibition of *CDKN1A* brought about a substantial diminished in the *BAX*/*BCL2* ratio (*p* ≤ 0.05) and *p53* (*p* ≤ 0.05) and *FAS* (*p* < 0.01) expression. Overexpression of *CDKN1A* induced a strong increase in the *BAX*/*BCL2* ratio (*p* < 0.01) and *p53* (*p* ≤ 0.05) and *FAS* (*p* < 0.01) expression. (**l**) Inhibition of *CDKN1A* promoted the production of E_2_ (*p* ≤ 0.05) and P_4_ (*p* < 0.01) by cumulus cells, and overexpression of *CDKN1A* inhibited the secretion of E_2_ and P_4_ (*p* < 0.01). (**m**) Inhibition of *CDKN1A* induced the expression of *STAR* (*p* < 0.01), *CYP11A1* (*p* < 0.01) and *HSD3B1* (*p* ≤ 0.05), and upregulation of *CDKN1A* expression inhibited *STAR* (*p* ≤ 0.05), *CYP11A1* (*p* < 0.01), and *HSD3B1* (*p* < 0.01) expression.

To elucidate the regulatory role of *CDKN1A* in DNA damage and steroid hormone secretion in bovine cumulus cells, *CDKN1A* was knocked down and overexpressed in the cells. After transfection of siCDKN1A-1, siCDKN1A-2, and siCDKN1A-3 into cumulus cells, RT–qPCR showed that the expression of CDKN1A was significantly decreased in the siCDKN1A-1 (*p* ≤ 0.05) and siCDKN1A-2 (*p* < 0.01) groups, while the expression was increased in the siCDKN1A-3 group (Figure 2a). Therefore, siCDKN1A-2 was used as a siRNA that specifically interferes with CDKN1Ain this paper. Next, *CDKN1A* overexpression plasmids were constructed. The results of RT–qPCR showed that the optimal treatment concentration was 500 ng/mL (*p* < 0.01), and the subsequent *CDKN1A* overexpression plasmids were treated with this concentration (Figure 2b). Figure 2c shows that inhibition of *CDKN1A* significantly attenuated the γH2AX-positive cell rate, and overexpression of *CDKN1A* significantly enhanced the γH2AX-positive cell rate (*p* ≤ 0.05). Moreover, remarkable changes in the expression of DNA damage-related genes and the expression of *BRCA1*, *MRE11*, and *ATM* were significantly increased after *CDKN1A* inhibition, while the expression of *RAD51* was markedly diminished (*p* ≤ 0.05). The promotion of *CDKN1A* expression resulted in suppression of *BRCA1*, *MRE11*, and *ATM* expression (*p* ≤ 0.05) and elevation of *RAD51* expression (*p* < 0.01). Cell cycle analysis by flow cytometry showed that the inhibition of *CDKN1A* blocked cell passage through the G1 (*p* ≤ 0.05) and S phases (*p* ≤ 0.05) and promoted rapid cell passage through the G2 phase (*p* ≤ 0.05, Figure 2e). The CCK-8 assay of proliferation levels after cell transfection showed that inhibition of *CDKN1A* significantly promoted proliferation (24 h, 48 h) and that overexpression of *CDKN1A* significantly inhibited cell proliferation levels (*p* ≤ 0.05, Figure 2f). The results of the RT–qPCR assay of cell cycle- and proliferation-related genes showed that knockdown of *CDKN1A* caused a significant increase in the expression of *CDK1* (*p* < 0.01), *CDK2* (*p* ≤ 0.05), and *CCNE2* (*p* ≤ 0.05) and a significant decrease in the expression of *CCND1* (*p* ≤ 0.05, Figure 2h). Overexpression of *CDKN1A* caused a marked diminish in the expression of *CDK1*, *CDK2*, and *CCNE2* (*p* ≤ 0.05) and a dramatic increase in the expression of *CCND1* (*p* < 0.01). Apoptosis assays showed that inhibition of *CDKN1A* substantially reduced the level of apoptosis (*p* ≤ 0.05) and that overexpression of *CDKN1A* induced apoptosis (*p* < 0.01, Figure 2g,i). The results of the apoptosis-related gene expression profile assay showed that knockdown of *CDKN1A* caused a drastic reduction in the *BAX*/*BCL2* ratio *(p* ≤ 0.05), *p53* (*p* ≤ 0.05) and *FAS* (*p* < 0.01), and overexpression of *CDKN1A* promoted the *BAX*/*BCL2* ratio (*p* < 0.01), *p53* (*p* ≤ 0.055), and *FAS* (*p* < 0.01, Figure 2j,k). ELISA results for E_2_ and P_4_ levels in cell cultures showed that E_2_ (*p* ≤ 0.05) and P_4_ (*p* < 0.01) levels increased significantly after *CDKN1A* inhibition, and overexpression of *CDKN1A* significantly promoted the secretion of E_2_ and P_4_ (*p* < 0.01, Figure 2l). Analysis of steroid hormone secretion-related gene expression showed that the knockdown of *CDKN1A* distinctly facilitated the expression of *STAR* (*p* < 0.01), *CYP11A1* (*p* < 0.01), and *HSD3B1* (*p* ≤ 0.05). In contrast, *CDKN1A* overexpression markedly inhibited the expression of *STAR* (*p* ≤ 0.05), *CYP11A1* (*p* < 0.01), and *HSD3B1* (*p* < 0.01, Figure 2m). 

### 3.3. miR-302d Targeted Binding to the CDKN1A 3′UTR and Inhibited Its Expression

**Figure 3:**
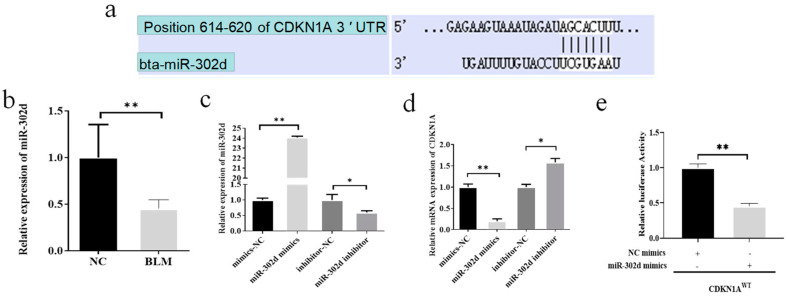
miR-302d targeted binding to the *CDKN1A* 3′UTR and inhibited its expression. Significant differences are denoted by * (*p* ≤ 0.05) and ** (*p* < 0.01). (**a**) Predictive discovery of miR-302d targeting binding to the *CDKN1A* 3′UTR. (**b**) BLM treatment of cumulus cells significantly inhibited miR-302d expression (*p* < 0.01). (**c**) Transfection of miR-302d mimics significantly increased miR-302d expression levels (*p* < 0.01), and the inhibitor significantly inhibited miR-302d expression (*p* ≤ 0.05). (**d**) Transfection of miR-302d mimics remarkably inhibited *CDKN1A* expression levels (*p* < 0.01), and the inhibitor significantly promoted *CDKN1A* expression (*p* ≤ 0.05). (**e**) Transfection with miR-302d mimics substantially lowered the relative activity of the wild-type reporter vector luciferase (*p* < 0.01).

To identify miRNAs that target and regulate *CDKN1A* during DNA damage in cumulus cells, this study utilized the TargetScan database (www.targetscan.org, accessed on 2 April 2023) in conjunction with data from earlier studies to screen miRNAs. TargetScan database predictions illustrate that miR-302d targets binding to the *CDKN1A* 3′UTR at positions 614 to 620 (Figure 3a). BLM treatment of bovine cumulus cells resulted in a significant down regulation of miR-302d expression (*p* < 0.01, Figure 3b), which was opposite to the trend of *CDKN1A* expression (Figure 1b) and was also in agreement with the results of earlier studies [24]. Transfection of bovine cumulus cells with miR-302d mimics enhanced miR-302d expression and significantly diminished *CDKN1A* expression (*p* < 0.01, Figure 3c,d). The miR-302d inhibitor dramatically inhibited miR-302dexpression while promoting *CDKN1A* expression (*p* ≤ 0.05). The dual luciferase reporter assay showed that miR-302d mimics remarkably inhibited the expression of *CDKN1A*^WT^ (Figure 3e). 

### 3.4. miR-302d Regulated DNA Damage and Steroid Hormone Secretion in Bovine CCs

**Figure 4:**
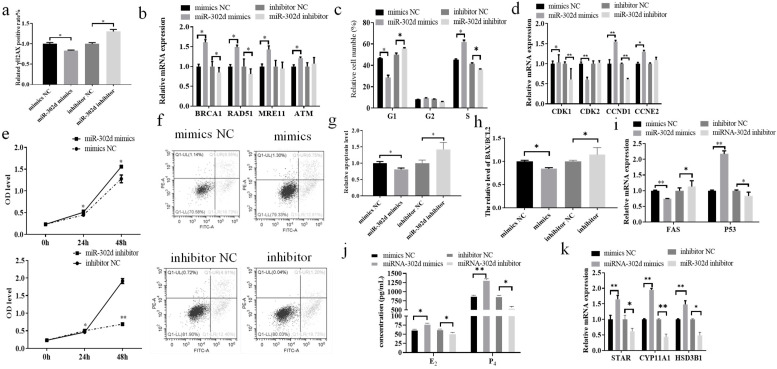
miR-302d regulated DNA damage and steroid hormone secretion in CCs. Significant differences are denoted by * (*p* ≤ 0.05) and ** (*p* < 0.01). Upon transfection of cells with miR-302d mimics or inhibitors, flow cytometry was employed to examine the rate of γH2AX-positive cells, cell cycle, and apoptosis, CCK-8 was used to detect cell proliferation, ELISA was used to analyze the content of E_2_ and P_4_ in the culture fluid, and expression levels of relevant genes were analyzed by RT–qPCR. (**a**) Transfection of mimics led to a marked decline in the rate of γH2AX-positive cells, and the inhibitor resulted in a significant increase (*p* ≤ 0.05). (**b**) The expression of *BRCA1*, *RAD51*, *MRE11*, and *ATM* was dramatically enhanced after transfection with miR-302d mimics (*p* ≤ 0.05); after transfection with miR-302d inhibitors, the expression of *BRCA1* and *RAD51* was greatly diminished (*p* ≤ 0.05), the expression of *MRE11* was decreased, and the expression of *ATM* was enlarged (*p* > 0.05). (**c**) A statistically significant decline in the number of cells in the G1 phase (*p* ≤ 0.05), a remarkable enlargement in the count of cells in the S phase (*p* ≤ 0.05), and an insignificant variation in the cell number in the G2 phase were caused by transfection with miR-302d mimics (*p* > 0.05). Upon transfection with the miR-302d inhibitor, the number of cells in the G1 phase was remarkably higher (*p* ≤ 0.05), the number of S phase cells was reduced considerably (*p* ≤ 0.05), and the number of G2 phase cells was diminished, but the effect of the difference was inconsequential (*p* > 0.05). (**d**) *CDK1* (*p* ≤ 0.05), *CCND1* (*p* < 0.01), and *CCNE2* (*p* < 0.01) expression was obviously elevated after transfection of miR-302d mimics and *CDK2* expression was notably diminished (*p* ≤ 0.05) in bovine CCs. Transfection of miR-302d inhibitor brought about a notable decline in *CDK1* and *CCND1* (*p* < 0.01), a rise in *CCNE2* expression, but the variety was insignificant (*p* > 0.05), and a rise in *CDK2* expression, but the variety was not obvious (*p* > 0.05). (**e**) miR-302d overexpression promoted cell proliferation at 24 h and 48 h (*p* ≤ 0.05), and inhibition of miR-302d interfered with cell proliferation at 24 h (*p* ≤ 0.05) and 48 h (*p* < 0.01). (**f**) The results of the apoptosis level assay after transfection of cells, left upper/Q1-UL: dead cells, right upper/Q1-UR: advanced apoptotic cells, right lower/Q1-LR: earlier apoptotic cells, left lower/Q1-LL: nonexpired cells. (**g**) Transfection of miR-302d mimics produced dramatic declines in relative apoptosis (*p* ≤ 0.05), and miR-302d inhibitor contributed to a noticeable improvement in the relative apoptosis levels (*p* ≤ 0.05). (**h**,**i**) *BAX*/*BCL2* expression was visibly displayed by transfection of the miR-302d mimic; the ratio of *BAX*/*BCL2* expression to that of *FAS* was visibly elevated by transfection of the miR-302d inhibitor, whereas the expression of *p53* was considerably minimized (*p* ≤ 0.05). (**j**) The levels of E_2_ and P_4_ were dramatically enlarged (*p* ≤ 0.05) after the miR-302d mimic was transfected, whereas the levels of E_2_ and P_4_ were diminished (*p* ≤ 0.05) after the miR-302d inhibitor was transfected. (**k**) The expression of *STAR*, *CYP11A1*, and *HSD3B1* was dramatically augmented after miR-302d mimic transfection (*p* < 0.01); the expression of *STAR* (*p* ≤ 0.055), *CYP11A1* (*p* < 0.01) and *HSD3B1* (*p* ≤ 0.05) prominently declined after transfection with miR-302d inhibitor.

In the present study, we clarified that miR-302d in bovine cumulus cells regulates the expression of *CDKN1A*. The expression of γH2AX in bovine CCs transfected with miR-302d mimics or inhibitors was examined by flow cytometry, and the results showed that the rate of γH2AX-positive cells was reduced by miR-302d overexpression, and inhibition of miR-302d promoted the γH2AX-positive cell rate (*p* ≤ 0.05, Figure 4a). The results of RT–qPCR detection of DNA damage-related genes showed that transfection of miR-302d mimics led to a pronounced increase in the expression of *BRCA1*, *RAD51*, *MRE11* and *ATM* in cells (*p* ≤ 0.05), while the inhibitor resulted in a distinct decrease in the expression of *BRCA1* and *RAD51* (*p* ≤ 0.05), a reduction in *MRE11* expression but with a nonsignificant difference (*p* > 0.05), and a slight but nonsignificant difference in the expression of *ATM* (*p* > 0.05, Figure 4b). miR-302d overexpression caused a noticeable decline in the number of cells in the G1 phase (*p* ≤ 0.05), a visible increase in the S phase (*p* ≤ 0.05), and an expansion in the number of cells in the G2 phase, but the difference was not significant (*p* > 0.05). Suppression of miR-302d drastically blocked the process of the G1 phase and caused cells to pass through the S phase rapidly (*p* ≤ 0.05), and the quantities of cells in phase G2 decreased, but the difference was not significant (*p* ≤ 0.05, Figure 4c). miR-302d overexpression led to an appreciable rise in the expression of *CDK1* (*p* ≤ 0.05), *CCND1* (*p* < 0.01) and *CCNE2* (*p* < 0.01) and a significant decrease in the expression of *CDK2* (*p* ≤ 0.05) in cumulus cells (Figure 4d). Inhibition of miR-302d resulted in a significant decrease in *CDK1* and *CCND1* (*p* < 0.01), an increase in *CCNE2* expression, but the variation was not marked (*p* > 0.05), and an increase in *CDK2* expression, but the difference was not obvious (*p* > 0.05). The results showed that overexpression of miR-302d significantly promoted cell proliferation (24 h and 48 h, *p* ≤ 0.05), and interference with miR-302d expression resulted in significant inhibition of proliferation (24 h (*p* ≤ 0.05), 48 h (*p* < 0.01), Figure 4e). Cell transfection with miR-302d mimics inhibited the relative level of apoptosis and inhibitor-induced apoptosis (*p* ≤ 0.05, Figure 4f,g). After cell transfection, RT–qPCR analysis of apoptosis-related genes showed that miR-302d overexpression dramatically reduced the *BAX*/*BCL2* ratio (*p* ≤ 0.05) and *FAS* expression (*p* < 0.01) and promoted *p53* expression (*p* < 0.01, Figure 4g–i). A decrease in miR-302d expression caused a significant elevation in the *BAX*/*BCL2* ratio and *FAS* expression and suppressed *p53* expression (*p* ≤ 0.05). ELISA analysis showed that an increase in miR-302d expression significantly promoted the generation of E_2_ (*p* ≤ 0.05) and P_4_ (*p* < 0.01), while a reduction in miR-302d expression was associated with a notable decline in the synthesis of E_2_ and P_4_ (*p* ≤ 0.05, Figure 4j). RT–qPCR detection of steroid hormone-related genes showed that miR-302d overexpression dramatically promoted the expression of *STAR*, *CYP11A1*, and *HSD3B1* (*p* < 0.01), and inhibition of miR-302d significantly interfered with the expression of *STAR* (*p* ≤ 0.05), *CYP11A1* (*p* < 0.01), and *HSD3B1* (*p* ≤ 0.05, Figure 4k).

## 4. Discussion

BLM-induced cellular DNA damage had an effect on the consequent production of γH2AX in a dose-dependent/time-dependent manner [28]. γH2AX not only indicates the extent of DNA damage but also serves as a signal transduction factor for DDR [29]. DNA damage in CCs causes COC communication disorder, which is dedicated to the inability of oocytes to initiate DDR, and increased DNA damage, bringing about the withdrawal of oocytes from meiosis [30,31]. DNA damage causes cell cycle arrest and induces apoptosis [10,11]. DNA damage has been linked to abnormal levels of estrogen and progesterone in women [13]. These results suggest that BLM causes DNA damage, induces apoptosis, arrests the cell cycle, and affects steroid hormone secretion. We found that the rate of γ-H2AX-positive cells increased significantly after BLM treatment, indicating that BLM successfully induced DNA damage in bovine cumulus cells. DNA damage in cumulus cells resulted in a significant decrease in cell abundance in the G1 phase and an obvious increase in cell abundance in the S phase, inhibited cell proliferation, increased relative apoptosis, and significantly reduced E_2_ and P_4_ secretion. During DNA damage, the expression of *CDKN1A* was apparently observed to expand, which was consistent with the results of previous studies [24], demonstrating that *CDKN1A* may be a key gene in the cellular DNA damage process.

DNA damage affects cellular physiological functions, cell lifespan depends on the effectiveness of DDR [32], and revitalization of the DDR pathway contributes to in vitro fertilization and embryo transfer [33]. *CDKN1A* inhibits DNA damage repair [21]. γH2AX is formed at DNA damage sites and is a biomarker of DNA damage that enhances cascading signaling during the DDR [26]. In this study, inhibition of *CDKN1A* resulted in a substantial decline in the positive rate of γH2AX in cells and changes in the expression of related DDR factors. DNA damage is able to activate ataxia-telangiectasia mutated (ATM), and once activated, ATM is phosphorylated and recruited to damaged DNA to initiate the DDR process [34]. Breast cancer susceptibility gene 1 (BRCA1) is a key member of the ATM-mediated DDR [35] and directs the homologous recombination repair (HR) pathway [36], and mutations in this gene are associated with ovarian cancers [37]. MRE11 promotes ATM activation, homologous recombination repair of DNA damage, and genome stability [38]. The expression of DNA damage repair-related genes is reduced [39], such as RAD51 [40], and the expression of RAD51 varies widely between species [41]. These findings support our hypothesis that inhibition of CDKN1A leads to a substantial expansion in *ATM*, *BRCA1*, and *MRE11* mRNA expression and a substantial reduction in *RAD51* expression. These changes activate the DDR pathway (HR pathway), reducing the γH2AX-positive rate and repairing DNA damage.

The stability and order of the cell cycle are foundations for the proliferation of cells and biological function performance. The phosphorylation of CDK2 and CCNE2 reaction products is related to the number of G1 phase cells [42]. Inhibition of CDKN1A leads to the upregulation of CDK2 and CCNE2, which enables CCs to pass through the G1 phase rapidly, promoting substance metabolism and RNA and protein synthesis. The interaction of CDKN1A and CDK1 leads to G2 phase abnormalities in the cell cycle [43]. Overexpression of CCND1 can lead to uncontrolled cell proliferation and corresponding cell cycle disorder in the G1 phase [44]. In this study, inhibition of CDKN1A altered the expression of cell cycle-related genes. Our study further confirmed that inhibition of CDKN1A led to up-regulation of CDK1 expression and down-regulation of CCND1 expression, which enabled a large number of cumulus cells to rapidly pass the G2 phase. The HR mechanism favors sister chromatids rather than homologous chromosomes as templates for DDR and is most active during the G2 phase when sister chromatids are available [45]. We hypothesize that inhibition of CDKN1A activates DNA damage repair through the HR pathway, leading to abnormal expression of cell cycle-related factors, leading to changes in cell cycle and proliferation. As a highly modulated program of cell death, apoptosis is a sensible and aggressive determination to compromise selected cells for the betterment of the biological organism and is a conventional and necessary physiological process in multicellular organisms [46]. Apoptosis plays an important role in cell renewal and embryonic development. Early studies confirmed that apoptosis is beneficial to multicellular organisms in a coordinated manner, allowing organisms to balance and fine-tune their life cycle [47]. Relevant studies have shown that CDKN1A allows DNA damage repair while inhibiting apoptosis, has the ability to activate apoptosis protein BAX to promote apoptosis, and can also promote apoptosis by relying on the p53 pathway [48]. Signals such as DNA damage and oncogene activation induce elevated levels of cellular *p53* and apoptosis [49]. In addition, FAS and FASL binding is also closely related to apoptosis [48,49,50], indicating that inhibition of *CDKN1A* activates the DDR mechanism and causes abnormal apoptotic gene expression, leading to declining apoptosis in cumulus cells.

DNA damage can lead to infertility and related endocrine disorders [13,51]. The abnormal secretion of estrogen and progesterone in cumulus cells results in low fecundity and weakened follicle development [52,53,54]. Downregulating 3β-hydroxysteroid dehydrogenase (HSD3B1) expression inhibited the synthesis of progesterone [55]. Cytochrome P450 family 11 subfamily A member 1 (CYP11A1) and steroidogenic acute regulatory protein (STAR) participate in cholesterol transport across mitochondrial membranes and cholesterol decomposition into pregnenolone, respectively [56]. In combination with BLM for cancer, abnormal expression of STAR, CYP11A1, and HSD3B1 led to hormone secretion disorder [55]. The secreted progesterone and estradiol of CCs are concerned with the expression of STAR, CYP11A1, and HSD3B1 [57,58]. In this study, the inhibition of CDKN1A significantly increased the mRNA levels of STAR, CYP11A1, and HSD3B1 in bovine CCs and significantly promoted the secretion of E_2_ and P_4_. These results indicated that CDKN1A could negatively regulate the secretion of steroid hormones in CCs. Therefore, the damage of CCs to estradiol and progesterone secretion caused by DNA damage may be caused by abnormal expression of steroid hormone marker genes. CDKN1A has become a focus of research on the regulatory mechanisms of reproduction as well as a critical factor in the post-transcriptional modulation of gene development [14]. miRNAs can affect DNA damage, proliferation, the cell cycle, and apoptosis by regulating target genes [15,59]. miRNA affected E_2_ and P_4_ secretion in CCs [16,17]. Disturbance of the pathway affected E_2_ and P_4_ synthesis and DNA damage in cumulus cells [52,60,61]. BLM treatment of CCs (Figure 3) caused a significant reduction in miR-302d expression, which was opposite to *CDKN1A* expression. miR-302d regulates the expression of *CDKN1A*. Combined with the results of the dual luciferase reporter gene, it was proven that miR-302d targeted *CDKN1A* and regulated its expression. In addition (Figure 4), the expression of *CDKN1A* in bovine cumulus cells transfected with miR-302d mimics decreased, the rate of γH2AX-positive cells decreased significantly, the cell cycle changed, proliferation was promoted, apoptosis was inhibited, and the secretion levels of E_2_ and P_4_ were increased. RAD51 can regulate the DDR through multiple pathways [62,63]. The expression of several targeted genes is controlled by a single miRNA [64]. The inconsistent expression of *RAD51* in cells transfected with miR-302d or siCDKN1A may be due to the change in the *RAD51* pathway to repair DNA damage or miR-302d regulation of other target genes to affect the expression of *RNA51*. miR-302d mimics upregulated *CCND1* expression and downregulated *CDK2* expression, causing a pronounced G2 phase cell count depression and an S phase cell count enlargement, which was inconsistent with the results of *CDKN1A* knockdown. It is possible that miR-302d regulates the expression of other genes involved in the cell cycle.

Oocyte quality is a key limiting factor for female fertility, but little is known about what constitutes oocyte quality or the mechanisms that control it. The developmental capacity of oocytes is mainly evaluated by the cumulus cell-mediated ovarian follicular microenvironment and the growth and development of oocytes [65]. DNA damage in cumulus cells is regarded as an important indicator for evaluating oocyte quality [66]. Different levels of cumulus cell DNA damage can reflect the developmental capacity of oocytes [67]. COCs coped with DNA damage better than denuded oocytes [8]. Our study found that inhibition of CDKN1A could activate the DDR pathway to reduce the degree of DNA damage in cumulus cells, suggesting that inhibition of CDKN1A could improve oocyte quality. The *p53* gene in cumulus cells can affect ovarian function by regulating the quality of mouse oocytes [68]. Specific inhibition of ATM may affect meiosis and cytoplasmic maturation in porcine oocytes by reducing their sensitivity to DNA damage [69]. In this study, after inhibiting *CDKN1A*, *p53* expression was downregulated and *ATM* gene expression was upregulated, which indicates that *CDKN1A* can improve oocyte quality and further regulate ovarian function by regulating the expression of cumulus cell genes.

Cumulus cells regulate the expression of hormonal factors and related regulatory factors in oocytes through the gap junction with oocytes, thereby affecting oocyte maturation [2]. DNA damage disrupts bovine COC communication, leading to oocyte withdrawal from meiosis [30], which in turn affects blastocyst production, hatchability, and embryo quality [70,71]. These results suggest that DNA damage in cumulus cells may affect the quality of oocytes and early embryos. Our study found that miR-302d targeted regulation of CDKN1A affected DNA damage and steroid hormone secretion in bovine cumulus cells. These results indicated that miR-302d and CDKN1A were candidate molecular markers for the diagnosis of DNA damage in bovine cumulus cells.

## 5. Conclusions

miR-302d targets *CDKN1A* and regulates its expression, resulting in reduced DNA damage levels, disordered cell cycle progression, increased proliferation levels, inhibited relative apoptosis levels, and increased steroid hormone secretion in bovine cumulus cells. Our current findings provided molecular targets for a more effective study of DNA damage in bovine cumulus cells.

## Data Availability

The data presented in this study are available in Supplementary Material.

## Supplementary Materials

The following supporting information can be downloaded at: https://www.mdpi.com/article/10.3390/genes14122195/s1, Table S1: Primer and siRNA sequences used in this study. Table S2: The raw data of Figure 1, Figure 2, Figure 3 and Figure 4.
