# Supplementary material for: miR-302d Targeting of CDKN1A Regulates DNA Damage and Steroid Hormone Secretion in Bovine Cumulus Cells
Source: Genes (Basel). 2023 Dec 10;14(12):2195. doi: 10.3390/genes14122195 (PMC10743266; doi:10.3390/genes14122195)
Supplement: Supplementary file 1 [file genes-14-02195-s001.zip › Table S1.pdf]

Table S1. Primer and siRNA sequences used in this study

Primers /siRNA	Sequence (5' to 3')[a]	Product size	GenBank accession Number	PCR Efficiency
FAS	F:AGCGAACTCCTGCCAAGAAG<br>R:TTCTGGTGTATCTCCGTCGC	110bp	NM_174662.2	106.50%
p53	F:GAATGTCCGAATGAAGCGCC<br>R:GAGGGGACAAAGGACGACAG	131bp	X81704.1	102.7%
BCL2	F:TGGCCTTCTTTGAGTTCGGA<br>R:GGCCATACAGCTCCACAAAG	181 bp	NM_214285	104.10%
BAX	F:GGCGGCTGAAATGTTTTCTGA<br>R:GTCCAATGTCCAGCCCATGAT	193 bp	NM_000633.3	101.80%
CDK1	F:CATTCGCCGCGGATAAAGC<br>R:CCACTTGGCCTGTAGTTTTGTG	152 bp	NM_001098958.1	103.23%
CDK2	F:GAGCCTCGGTTGCATCTTTG<br>R:CCACTTGGGGAAACTTGGCT	131 bp	NM_001014934.1	98.61%
CCND1	F:AACGGCTTCCTCTCCTATC<br>R:TCCTCCTCCTCCTCTTCC	145bp	NM_001046273.2	105.11%
CCNE2	F:GCTGCTGCTGCCTTATGC<br>R:TCTGTATGTTGTGTCTGTCTTCC	107bp	XM_025001684.1	98.15%
RAD51	F:CGCCCAAGATGACCACCAC<br>R:TGGTGGAATTTGGAGCTGGG	134bp	NM_001046179.2	102.70%
BRCA1	F:GCAGAGTGAACCTGAAAATCCT<br>R:TGTGTCTGCTGCTTTGTCCT	158bp	NM_178573.1	96.65%

ATM	F:GCCGTGATGACCTGAGACAAGATG<br>R:AGAACGCCACTTCGCTGAGAAAG	144bp	NM_001205935.1	94.08%
MRE11	F:CGCTTCCGCCTTGACTGCTG<br>R:TCTGCCCTCTTCCTCCTCTTTGG	135bp	XM_024975290.1	100.90%
GAPDH	F:ACCCAGAAGACTGTGGATGG<br>R:ATGCCTGCTTCACCACCTTC	247bp	NM_001034034.2	108.90%
CDKN1A	F:GCAUGACAGAUUUCUACCATT<br>R:UGGUAGAAAUCUGUCAUGCTT	104bp	NM_001098958.2	107.23%
STAR	F:CTGAGCAGATCATGAAGACAGG<br>R:GTCCAGTTCATCTCCAATGCG	104bo	NM_174189.3	100.90%
CYP11A1	F:ACTGAGTACCTGAACCGGCA<br>R:CACTTATGGCCCAGATAGGCA	147bp	NM_176644.2	95.88%
HSD3B1	F:GCCACTGGATGGCGAGTATT<br>R:AGGGAGACTGCCCCTTCTTA	200bp	NM_174343.3	107.00%
U6	F:GCTTCGGCAGCACATATACTAAAAT<br>R:CGCTTCACGAATTTGCGTGTCAT<br>RT:CGCTTCACGAATTTGCGTGTCAT			106.10%
miR-302d	F:GCGCGTAAGTGCTTCCATGT<br>R:AGTGCAGGGTCCGAGGTATT<br>RT:GTCGTATCCAGTGCAGGGTCCGAGGTATTCGCACTGGATACGACACT AAA			99.98%
siCDKN1A-1	Sense-GCGGUGGAACUUCGACUUUTT<br>Antisense-AAAGUCGAAGUUCCACCGCTT			
siCDKN1A-2	Sense-GCAUGACAGAUUUCUACCATT			

	Antisense-UGGUAGAAAUCUGUCAUGCTT
siCDKN1A-3	Sense-GCAGACUGAUCUGCUCCAATT
	Antisense-UUGGAGCAGAUCAGUCUGCTT
siRNA-NC	Sense-UUCUCCGAACGUGUCACGUTT
	Antisense-ACGUGACACGUUCGGAGAATT

[a] F: Forward primer; R: Reverse primer; RT: Reverse transcription primer.
